# Defending one's friends, not one's enemies: A social network analysis of children's defending, friendship, and dislike relationships using XPNet

**DOI:** 10.1371/journal.pone.0194323

**Published:** 2018-05-18

**Authors:** Beau Oldenburg, Marijtje Van Duijn, René Veenstra

**Affiliations:** Interuniversity Centre for Social Science Theory and Methodology, Department of Sociology, University of Groningen, Groningen, the Netherlands; Tampere University of Technology, FINLAND

## Abstract

Previous studies investigating to what extent students in elementary schools defend their victimized classmates typically treated defending as an individual characteristic. Defending should, however, be seen as a directed dyadic relationship between a victim and a defender, who are embedded multiple positive and negative relationships with each other and their classmates. Accordingly, in the present study defending was investigated using social network analysis. More specifically, it was investigated to what extent defending relationships co-occurred with friendship and dislike relationships involving not only the victim and the defender but also other classmates. Bivariate Exponential Random Graph Models (ERGMs) were used to analyze the defending-friendship and defending-dislike relationships in seven grade-three classrooms. As hypothesized, the results indicated that victimized students were likely to be defended by students who they perceive as friends or who perceive them as friends. Moreover, defending was likely to occur when the victim and (potential) defender had the same friends. Victimized students were unlikely to be defended by classmates whom they disliked or who had indicated to dislike them. Finally, defending was likely to occur between students who disliked the same classmates.

## Introduction

School bullying, the systematic and intentional abuse of students who cannot easily defend themselves, is a common problem in schools all over the world [1]. Being victimized is linked to numerous negative outcomes, varying from physical injuries to psychosocial maladjustment (e.g., anxiety and depression) [2, 1]. The harmful effects of bullying can continue long after the actual bullying took place [3]. One important reason for why students bully is that they aspire to social status in the peer group [4, 1]. By harassing others, bullies aim to demonstrate their power to the rest of the group [5]. Accordingly, bullying nearly always occurs in the presence of witnessing peers [6, 7].

Students who witness that their classmates are being bullied can react to this in three ways. They can: 1) support the bullying (e.g., join in or cheer), 2) ignore the bullying (e.g., walk away from it or pretend not to see it), or 3) defend the victim (e.g., help or comfort the victim). By supporting or ignoring the bullying, witnessing students inadvertently signal to the bully and victim that the bullying is ‘cool’ or that it at least is acceptable behavior. Conversely, by defending victimized classmates, students signal that they do not accept or like this kind of behavior. When most students disapprove the bullying and defend the victim, bullying is no longer an effective strategy to climb the social ladder. Indeed, one study demonstrated that defending was negatively associated with the frequency of bullying in the classroom [8]. In addition, defending victimized classmates potentially mitigates the negative effects of bullying: one (cross-sectional) study demonstrated that defended victims had a better psychosocial adjustment than undefended victims [9].

### Investigating defending using social network analysis

Defending is thus important: it may alter the bully’s behavior and can provide a buffer against the negative consequences of bullying. However, students rarely defend their victimized classmates [10]. In the past decade several studies sought to better understand why this is the case. Although these studies provided valuable insights, nearly all of them focused on individual characteristics of defenders [11,12], hereby ignoring that defending actually is a relational phenomenon. That is, defending is a directed dyadic relationship in which by definition at least two actors (i.e., a victim and a defender) are involved. This implies that rather than being a defender (i.e., always behaving in this way), students’ behavior can be flexible; they may defend certain classmates but remain passive when other classmates are victimized [13].

We argue that in order to take this relational nature of defending into account, defending behavior should be investigated using social network analysis. Although social network analysis has recently been used to investigate various types of positive and negative relationships among primary school and high school students (e.g., helping, liking, and bullying relationships) [14–16], only two studies have used social network analysis to investigate defending behavior. The first study, a study by Sainio and colleagues [9], investigated defending by analyzing dyadic relationships between victims and (potential) defenders. The second study, a study by Huitsing and colleagues [15], carried the analyses of Sainio and colleagues a step further and investigated to what extent defending networks co-evolved with victimization and bullying networks. Huitsing and colleagues found that over time victims of the same bullies defended each other, defenders ran the risk of becoming victimized by the bullies of the victims they defended, bullies with the same victims defended each other, and defenders of bullies joined the harassment of those bullies’ victims.

Apart from these insights, not much is known about defending relationships and defending networks. Therefore, the aim of the present study was to further investigate defending networks in Dutch elementary schools. We were particularly interested in examining to what extent defending relationships co-occurred with two common types of positive and negative relationships among elementary school students: friendship and dislike.

#### Friendship

Nearly all children and adolescents are involved in friendships [17]. It is therefore not surprising that one of the first studies on students’ social relationships was on friendship relationships [18]. Being friends with someone entails more than simply liking this person; friendship implies a certain degree of responsibility for each other’s well-being. Consistent with this, several studies found a clear link between friendship and helping [19–22]. It is likely that students feel responsible for defending their victimized friends and also expect to be defended by their friends when they are bullied.

A complication—and a possible explanation for why defending and friendship relationships may not fully overlap—is that friendships are not necessarily reciprocal. Although it is commonly assumed that when student *i* perceives student *j* as a friend, *j* will also perceive *i* as a friend, research suggests that this is not always the case [23].

In this study, three different friendship variations could be observed: 1) friendships in which the victim nominated the defender as a friend, but the defender did not reciprocate this nomination, 2) friendships in which the defender nominated the victim as a friend, but the victim did not reciprocate this nomination, and 3) reciprocated friendships. Accordingly, we did not only investigate to what extent defending relationships co-occurred with reciprocal friendship relationships, but also investigated to what extent defending co-occurred with unreciprocated friendship relationships.

Sainio and colleagues [9] focused on dyadic defending relationships. However, relationships do not occur in isolation, but are interdependent [24]. In other words, the presence (or absence) of certain relationships may affect the presence (or absence) of other relationships. For instance, according to Heider’s [25] balance theory, individuals tend to befriend friends of their friends. We argue that students are likely to defend victimized friends of their friends because even though they are not directly friends with these students (yet), they may have positive feelings towards them. In addition, by defending a victimized friend of their friend students may do this friend a favor.

#### Dislike

Hembree and Vandell [26] demonstrated that dislike relationships are like friendship a common occurrence during childhood: a large majority of the students in their study had at least one (mutual) dislike relationship. We argue that defending relationships are unlikely to co-occur with dislike relationships. Research demonstrated that students who defend victimized classmates run the risk of becoming targets of bullying as well [15, 27, 12]. We argue that students are unlikely to be willing to face this risk for victims whom they dislike. Accordingly, we expected that defending was unlikely to occur when the (potential) defender disliked the victim.

Similarly to defending, in most previous studies dislike has been treated as an individual construct (i.e., rejection) rather than as a relational construct [28, 29]. However, students may have dislike relationship with some classmates but not with others. In addition, similarly to friendship relationships, dislike relationships may be asymmetric. Accordingly, we investigated to what extent unreciprocated dislike relationships co-occurred with defending relationships.

In addition to dyadic dislike relationships, we investigated dislike relationships in groups. Heider’s [25] balance theory does not only imply that ‘friends of my friends are my friends’ but also that ‘enemies of my enemies are my friends’. Being disliked by the same classmate or disliking the same classmate may create a bond. Therefore, we expected that defending relationships were likely to occur between students who were disliked by the same classmates or between students who disliked the same classmates.

### The present study

In short, the present study aimed to contribute to prior studies on defending behavior by investigating defending networks in Dutch elementary schools. We investigated to what extent defending relationships co-occurred with two common types of positive and negative relationships among elementary school students: friendship and dislike. We hypothesized that defending was likely to occur between friends (hypothesis 1) and between friends of friends (hypothesis 2). In addition, we hypothesized that defending was unlikely to co-occur with dyadic dislike relationships (hypothesis 3). Finally, we hypothesized that defending relationships were likely to occur between students who were disliked by the same classmates (hypothesis 4) or between students who disliked the same classmates (hypothesis 5).

## Method

### Sample and procedure

The data were part of a larger ongoing project evaluating the effectiveness of the Dutch version of the KiVa anti-bullying program [15, 30, 31]. All data reported here were collected in May 2012, before the KiVa program was implemented. The data consisted of 462 elementary school classrooms. In order to be able to directly compare the parameter estimates of the different classrooms, only (single grade) grade 3 classrooms with the median number of students (i.e., 23) were selected. The selected sample consisted of 7 classrooms.

Participating students filled out web-based questionnaires during regular school hours. The students read the questionnaire by themselves; difficult concepts were explained in instructional videos. In these videos a professional actress explained the questions in such a way that students would understand them (e.g., by using age-appropriate language, talking slowly, and articulating words clearly). Classroom teachers were present to answer questions and to assist students whenever necessary. Teachers were supplied with detailed instructions before the data collection had started and they were encouraged to help students in such a way that it would not affect their answers (e.g., by asking them questions such as “Which words are unclear to you?”).

### Ethics statement

Two of the authors were responsible for the data collection. Observational research using data does not fall within the ambit of the Dutch Act on research on human subjects and does not need approval of an ethics committee. The data were anonymized before the analyses, and questionnaires were completed on a voluntary basis. Schools sent a letter with information about the study’s aims and procedures and a permission form to the students’ parents before the data were collected. Parents who did not want their child to participate in the assessment were asked to return the form. Students were informed at school about the research and gave oral consent. Students were reassured that their answers would remain confidential and they were advised not to talk about their answers to others. Both parents and students could withdraw from participation at any time. Students who did not receive parental consent, or who did not want to participate themselves, or who were unable to fill in the questionnaire, did not participate (1.5%). The main reason for this high response rate is that data were collected online and teachers were informed about which of their students filled in the questionnaire. Moreover, students who incidentally missed the scheduled day of data collection could participate on another day within a month.

### Defending, friendship, and dislike networks

#### Defending networks

The defending networks were measured by asking victimized students which classmates defended them. Students who had experienced at least one form of bullying (e.g., physical, relational, or material bullying) in the past months were asked to nominate their defenders. Victimization was measured using the Olweus bully/victim questionnaire [32]. Victimized students read a description in which it was explained that some children help others who are bullied by supporting them, comforting them, or by telling the bullies to stop. After they read the description, the victimized students were asked to select the names of classmates who supported, comforted, or helped them when they were bullied. Victims could select an unlimited number of classmates as defenders. The defending variable had score 1 when victim i nominated classmate j as a defender (i.e., there was a defending relationship) and score 0 when victim i did not nominate classmate j as a defender (i.e., there was no defending relationship). By measuring defending in this way, defending a victimized classmate was represented by an incoming nomination. In other words, defenders were nominated by the victims whom they defended.

#### Friendship and dislike networks

Students were asked to select the names of classmates who were their best friends and of classmates whom they disliked. They could select an unlimited number of classmates for both questions. The friendship and dislike variables had score 1 when student i nominated classmate j (i.e., there was a friendship/dislike relationship) and score 0 when student i did not nominate classmate j (i.e., there was no friendship/dislike relationship).

### Gender

Several studies demonstrated that during childhood and early adolescence social interaction predominantly takes place in same-gender peer groups [33–35]. Moreover, helping relationships are more likely to occur in girls’ relationships than in boys’ relationships [36, 37, 16]. Accordingly, we added configurations reflecting gender similarity and gender sender and receiver effects to the models. Boys were coded as 1. The number of boys ranged from 9 to 13 boys per classroom.

## Analyses

The hypotheses were tested using Exponential Random Graph Models (ERGMs, for a comprehensive overview see [38, 39]). ERGMs allow the researcher to investigate which patterns (e.g., reciprocity or transitivity) characterize relationships in an observed social network. Based on theoretical arguments, the researcher selects network configurations which are included in the model as ‘explanatory variables’. Somewhat similar to logistic regression, a positive parameter indicates a higher occurrence of the configuration in the observed network than would be expected by chance. In ERGMs the interpretation of every model parameter is conditional on the other parameters in the model. This implies that dyadic configurations need to be taken into account when interpreting triadic or higher order configurations.

We used XPNet [40] to analyze the defending-friendship networks and defending-dislike networks. XPNet is the only software available for the estimation of multivariate ERGMs (for a comprehensive overview see e.g., [41]). The maximum number of networks that can be analyzed simultaneously in XPNet is two.

In ERGMs univariate configurations are used as building blocks for the bivariate models. We used the configurations of Huitsing and colleagues ([14], friendship and dislike) and Huitsing and Veenstra ([13], defending) as starting points for selecting univariate defending, friendship, and dislike configurations. Based on the XPNet user manual [40] bivariate configurations that best matched the hypotheses were selected.

**Fig 1:**
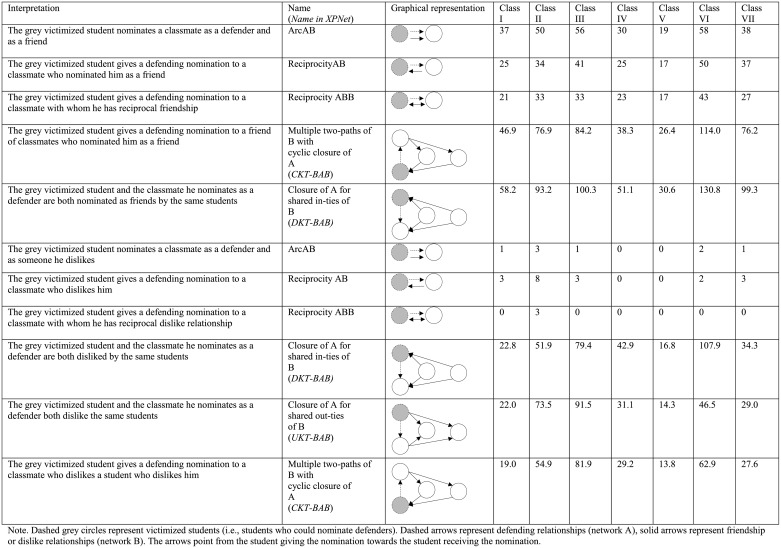
Configurations defending-friendship and defending-dislike.

Fig 1 provides an overview of the selected bivariate configurations. Similarly to the studies of Sainio and colleagues [9] and Huitsing and colleagues [15], in this study victimized students were asked who defended them. It is important to note that by measuring defending in this way, defending a victimized classmate was represented by an incoming nomination. The dashed grey circles in Fig 1 represent victimized students (i.e., students who could nominate defenders). Dashed arrows represent defending relationships (network A), solid arrows represent friendship or dislike relationships (network B). The arrows point from the student giving the nomination towards the student receiving the nomination. The seven classrooms are indicated by Roman numerals.

For the defending-dislike networks it was not possible to include the reciprocityABB configuration in the model because, as expected, this configuration was (almost) never observed in the selected classrooms (see Fig 1). In our sample victimized students were unlikely to give defender nominations to classmates with whom they had a reciprocated dislike relationship.

In order to take into account that only victimized students could nominate defenders, non-victimized students were treated as structural zeros in the defending networks. These students could receive defender nominations but could not give defender nominations. The number of students who were treated as structural zeros ranged from 4 to 12 per classroom.

XPNet uses Markov Chain Monte Carlo (MCMC) methods to obtain Maximum Likelihood estimates. The model estimation successfully converges when the values of all t-statistics of parameters that are included in the model are smaller than 0.10 [40]. As suggested by Wang, Robins and Pattinson [40] we increased the multiplication factor for models that did not converge using the default settings. The used settings are listed in the footnotes of the tables displaying the results of the analyses. Once all t-statistics had reached a value smaller than 0.10, XPNet calculated the goodness of fit statistics. The model fitted the data when (most) t-statistics of parameters that were not included in the model were lower than 2. Through careful parameterization an acceptable goodness of fit was obtained for all networks.

In order to be able to draw general conclusions, the results of the two analyses were combined in two meta-analyses, using R-package metafor [42]. Due to the selection of classrooms with the same size, the parameter estimates can be considered as parallel measures and are therefore comparable on the same scale. Average parameter estimates with standard errors were obtained in the meta-analysis, facilitating an overall test of the hypotheses. Moreover, the meta-analyses indicated whether the estimates varied significantly over the seven classrooms.

## Results

Table 1 displays the density (i.e., the relative number of relationships in the network) and the reciprocity of the defending, friendship, and dislike networks. Overall, the density and reciprocity were highest in the friendship networks, and lowest in the defending networks.

**Table 1:** Density and reciprocity.

	I	II	III	IV	V	VI	VII
Density defending	0.10	0.13	0.14	0.07	0.04	0.15	0.10
Reciprocity defending	0.13	0.14	0.16	0.17	0.10	0.17	0.19
Density friendship	0.21	0.25	0.26	0.22	0.25	0.34	0.38
Reciprocity friendship	0.23	0.30	0.27	0.29	0.33	0.32	0.23
Density dislike	0.11	0.22	0.25	0.25	0.16	0.17	0.19
Reciprocity dislike	0.16	0.21	0.31	0.31	0.18	0.17	0.20

### Defending-friendship networks

**Fig 2:**
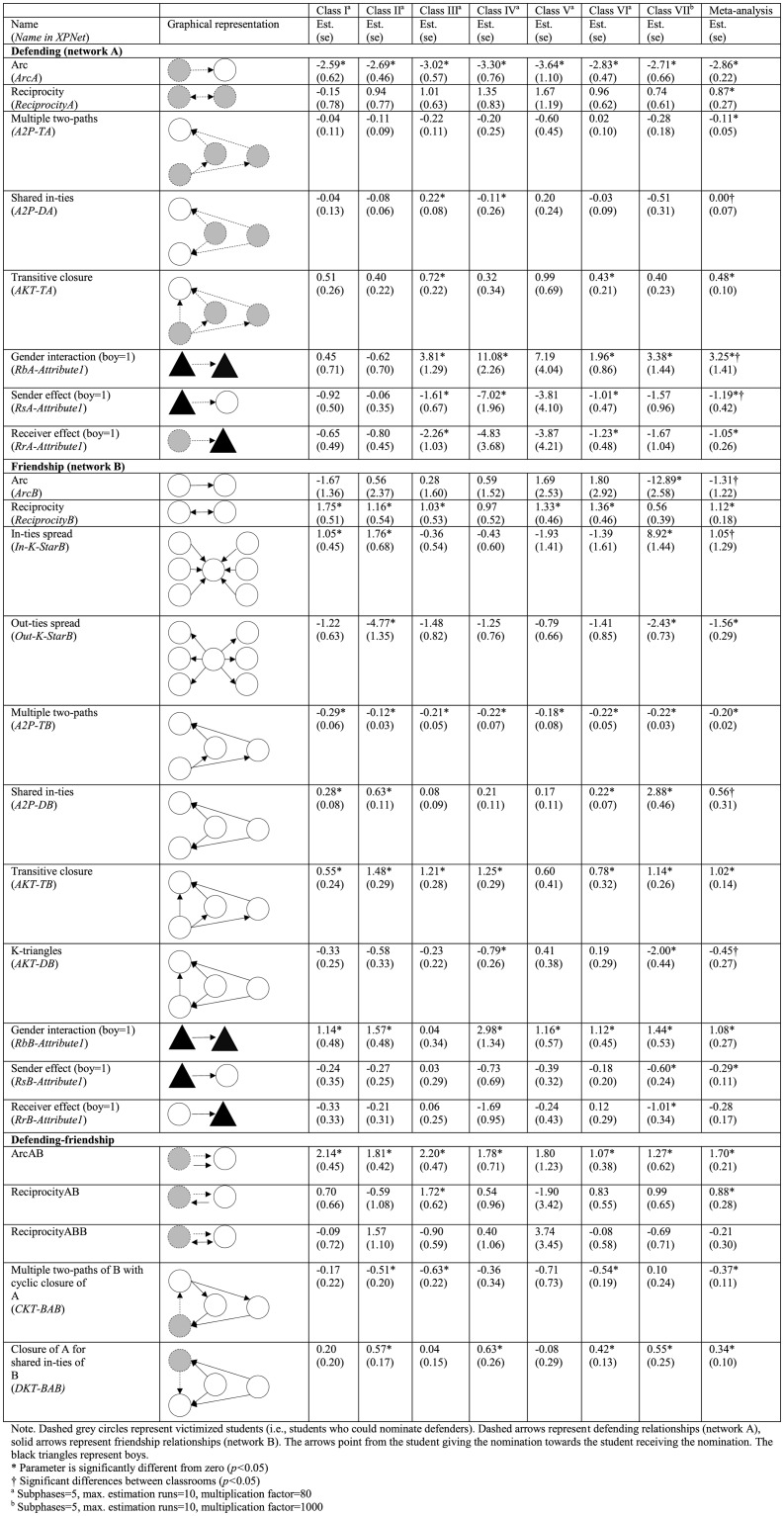
Defending-friendship.

Fig 2 displays the results of the analysis of the defending (network A) and friendship (network B) networks. Again, dashed grey circles represent victimized students (i.e., students who could nominate defenders). Dashed arrows represent defending relationships (network A), solid arrows represent friendship (network B). The arrows point from the student giving the nomination towards the student receiving the nomination.

The univariate statistics demonstrate that defending (*b* = 0.87, *p*<0.001) and friendship (*b* = 1.12, *p*<0.001) were likely to be reciprocated. Moreover, the defending networks were characterized by clustering rather than connectivity (multiple two-paths, *b* = -0.11, *p* = 0.03; transitive closure, *b* = 0.48, *p*<0.001). The friendship networks also exhibit various clusters (out-ties spread, *b* = -1.56, *p*<0.001; multiple two-paths, *b* = -0.20, *p*<0.001; transitive closure, *b* = 1.02, *p*<0.001). Moreover, Fig 2 demonstrates that defending and friendship often occurred in same-gender groups (gender interaction defending, *b* = 3.25, *p* = 0.02; gender interaction friendship, *b* = 1.08, *p*<0.001).

The meta-analysis indicates that classrooms differed significantly from each other in the size of the gender similarity effect for defending. For instance, in classrooms I, II, and V no significant effects were found, whereas in classrooms III, IV, VI, and VII strong effects were found. In fact, the defending network in classroom IV was completely segregated, which explains the high parameter estimates in this classroom.

We hypothesized that defending was more likely to occur between friends and investigated three dyadic friendship variations. We found that victimized students were indeed likely to give defending nominations to students who they also nominated as their friend (arcAB, *b* = 1.70, *p*<0.001). In addition, we found that victimized students were likely to give defending nominations to students who nominated them as a friend (reciprocityAB, *b* = 0.88, *p* = 0.002). The reciprocity ABB configuration can be interpreted as a combination of the arc AB and reciprocity AB configurations. Fig 2 shows a small non-significant negative estimate, indicating that there is no additional effect of this specific configuration.

In addition, we hypothesized that defending was likely to occur between friends of friends. Taking into account the other effects in the model, we found that defending was more likely to occur when the victim and potential defender were both nominated as a friend by other classmates (closure of A for shared in-ties of B, *b* = 0.34, *p*<0.001). Victimized students were unlikely to give defending nominations to friends of classmates who nominated them as friends (multiple two-paths of B with cyclic closure of A, *b* = -0.37, *p* < .001), indicating that there is not a tendency toward generalized exchange but to a non-cyclic (hierarchical) ordering.

### Defending-dislike networks

**Fig 3:**
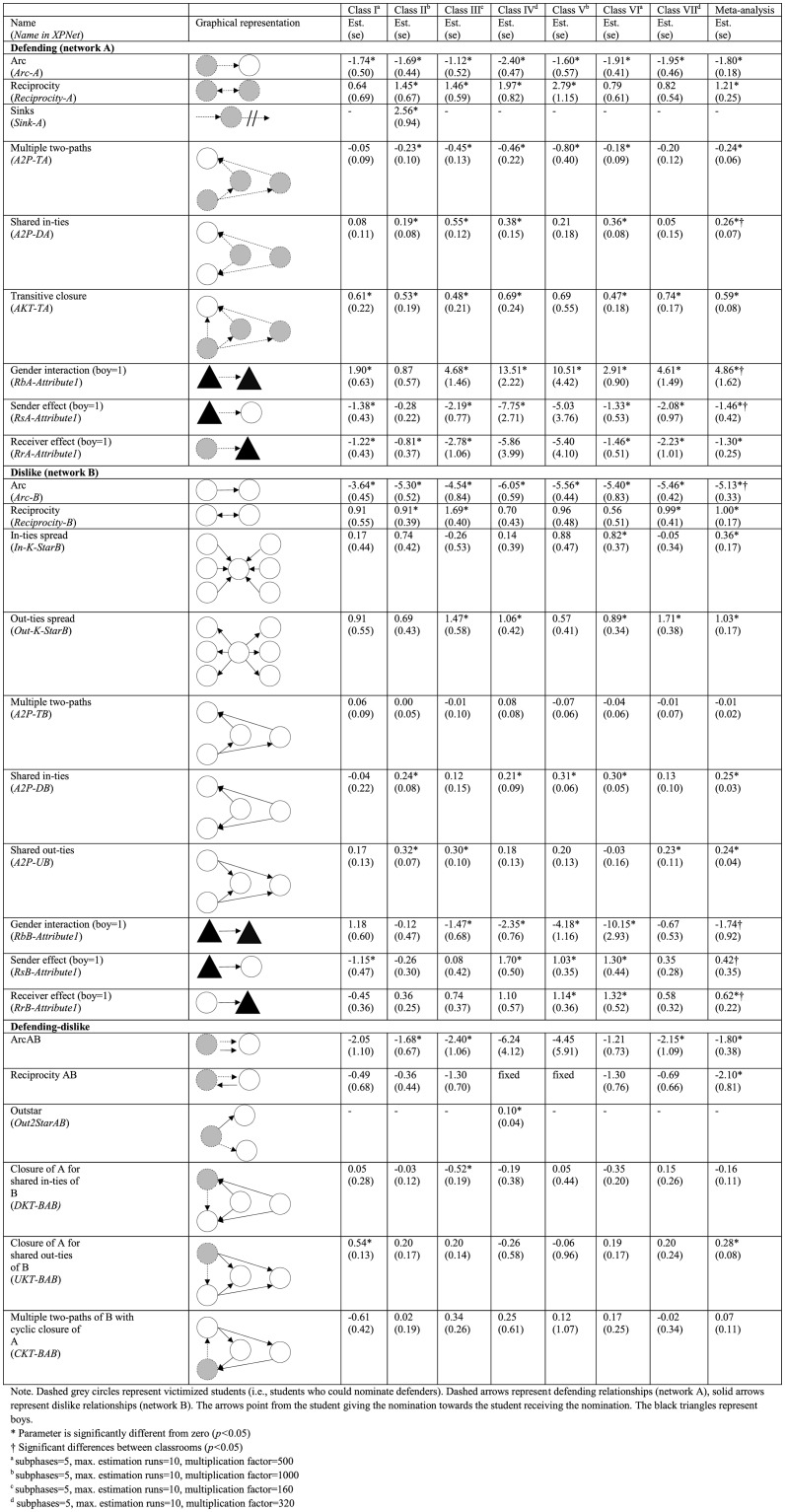
Defending-dislike.

Fig 3 displays the results of the analysis of the defending (network A) and dislike (network B) networks. In order to obtain converged models with an acceptable goodness of fit, a sink configuration was added for the defending network of classroom II and an out-star configuration was added for the defending-dislike network of classroom IV. The univariate statistics show that dislike nominations were likely to be reciprocated (*b* = 1.00, *p*<0.001). Moreover, students differed in the number of dislike nominations they received from (in-ties spread, *b* = 0.36, *p* = 0.04) and gave to classmates (out-ties spread, *b* = 1.03, *p*<0.001). Students were likely to be disliked by the same classmates (shared in-ties, *b* = 0.25, *p*<0.001) and to dislike the same classmates (shared out-ties, *b* = 0.24, *p*<0.001).

We expected that defending was unlikely to co-occur with dyadic dislike relationships. Consistent with this hypothesis, we found that it was unlikely that victims gave defender nominations to classmates whom they disliked (arcAB, *b* = -1.8o, *p*<0.001) and to classmates who disliked them (reciprocityAB, *b* = -2.10, *p* = 0.01). Note that the reciprocityAB parameters of classrooms IV and V had to be constrained in order to obtain converged models.

We hypothesized that defending relationships were likely to occur between students who were disliked by the same classmates, but did not find support for this hypothesis. Finally, as expected we found that defending was likely to occur between students who disliked the same classmates (closure of A for shared out-ties of B, *b* = 0.28, *p*<0.001).

## Discussion

Defending is important: it may alter the bully’s behavior and can provide a buffer against the negative consequences of bullying. The present study aimed to contribute to prior studies on defending behavior by investigating to what extent defending relationships co-occurred with two common types of positive and negative relationships among elementary school students: friendship and dislike.

We argued that students are likely to feel responsible for helping their victimized friends and predicted that defending was likely to occur between students who were friends. We investigated three dyadic friendship variations. The analyses showed that victimized students were indeed likely to give defending nominations to students who they also nominated as their friend. Moreover, we found that victimized students were likely to give defending nominations to students who nominated them as friend.

Consistent with balance theory [25] we found that students were likely to defend the defenders of their defenders and befriend the friends of their friends. We hypothesized that defending was likely to occur between friends of friends and found that defending was more likely to occur when the victim and (potential) defender were both nominated as a friend by other classmates. However, we also found that victimized students were unlikely to give defending nominations to friends of classmates who nominated them as friends. An explanation for this finding is that in positive networks, there is a tendency to have a hierarchical ordering with relatively little cyclic closure. For that reason, longitudinal social network studies using the SIENA software often find a negative estimate for the three-cycle parameter [35].

We expected that defending was unlikely to co-occur with dyadic dislike relationships. Similarly to the investigation of friendship, we investigated not only reciprocated but also unreciprocated dislike relationships. As expected, we found that it was unlikely that victims gave defender nominations to classmates whom they disliked. In addition, victimized students were unlikely to give defender nominations to classmates who disliked them. Moreover, as the descriptive statistics showed, the victimized students in our sample did not give defender nominations to classmates with whom they had a reciprocated dislike relationship.

Finally, we expected that defending relationships were likely to occur between students who were disliked by the same classmate but did not find support for this hypothesis. We did find that defending was likely between students who disliked the same classmates. It may be that these disliked classmates are the victims’ bullies. This explanation is in line with the study of Huitsing and colleagues [15] who found that victims of the same bullies defended each other.

Consistent with previous studies demonstrating that during childhood and early adolescence social interaction predominantly takes place in same-gender peer groups [33–35], we found that defending often occurred in same-gender groups. The strength of the gender effects varied per classroom. That is, in some classrooms no gender effects were found, whereas in other classrooms the defending network was completely segregated. In a larger study classroom gender effects might be further studied.

The findings of this study should be interpreted tentatively. We hope that future studies will repeat our study using a larger sample and students of different age groups. Future studies may also further investigate differences in defending between classrooms. Moreover, given that defending was measured by reports of victims, it is unclear whether students were actually defended by the classmates whom they nominated as defenders. Although, students were asked to report on actual defending, it is possible that victimized students nominated their friends as hypothetical defenders, even though they had not actually been defended by these classmates. In other words, students may have nominated friends whom they perceived as potential defenders. Our data did not allow to test whether the nominated defenders confirmed their behavior. Even though it is the perceived defending rather than the *actual* defending that affects the victim’s well-being [9], it would be interesting to find out whether victims and the students they nominate as defenders agree on their defending relationship.

Furthermore, a rather broad definition of defending behavior was used in this study. Defending was defined as helping, supporting, or comforting the victim. It would be interesting to investigate possible differences between publicly standing up for the victim and more subtle forms of defending behavior, such as comforting the victim after the bullying. In addition, in most studies on school bullying students are considered victimized when they had been bullied at least twice a month, whereas in the present study students were considered victimized when they had been bullied at least once in the past four months.

Despite these limitations, the present study can be considered a first step in investigating defending, friendship, and dislike relationships using a social network approach. We conclude that victimized students were indeed likely to give defending nominations to students who they also nominated as their friend or who nominated them as friend. Moreover, defending was more likely to occur when the victim and potential defender were both nominated as a friend by the same classmates. In addition, we conclude that victims were unlikely to give defender nominations to classmates whom they disliked or who had indicated to dislike them. Finally, we found that defending was likely to occur between students who disliked the same classmates, perhaps the bullies. Given that the strength of some patterns varied per classroom, it seems that classroom characteristics affect defending behavior as well. This may imply that when addressing defending behavior, for instance by anti-bullying interventions, uniform measures may not be adequate. We hope that future studies will follow up on our study and further investigate this.

## Supporting information

S1 Defending Text files with the defending networks of the seven classrooms. (ZIP)

S2 Dislike Text files with the dislike networks of the seven classrooms. (ZIP)

S3 Friendship Text files with the friendship networks of the seven classrooms. (ZIP)

S4 Gender Text files with the gender of the students in the seven classrooms (boy = 1). (ZIP)

S5 Structural zeros Text files indicating non-victimized students who could not give defender nominations and were treated as structural zeros (structural zero = 1). (ZIP)
